# The International Homœopathic Congress

**Published:** 1891-03

**Authors:** Pemberton Dudley

**Affiliations:** Sec. of the Com. and Genenal Secretary of the A. I. H.; S. W. Cor. 15th and Master Sts., Philadelphia, Pa.


PEMBERTON DUDLEY, M. D.,

*General Secretary, A. I. H.*

S. W. COR. 15TH AND MASTER STS.,
PHILADELPHIA, PA.

---

### THE INTERNATIONAL HOMŒOPATHIC CONGRESS.

EDITORS OF THE HOMŒOPATHIC PHYSICIAN:—The American Institute's Committee on the International Homœopathic Congress is endeavoring to give direction and character to the Essays and Discussions of the Congress, and to this object more time and energy have been devoted than to any other part of the Committee's labors. It would seem that as the themes and discussions of a national medical association naturally take a broader scope than those of a local society, so the work of an International Congress should be more comprehensive and far-reaching than even that of a national convention. This Committee is, therefore, seeking to bring before the approaching Congress some of the highest and broadest questions that confront our profession in all its departments. It is important that the Congress should discuss, for instance, some of the broad and imperative issues of modern Surgery, rather than the technical details of some minor or major operation—the influence of the Law of Cure in a whole realm of maladies, rather than the indications for this or that remedy in some particular disease—the construction and promulgation of a materia medica, rather than the symptoms of an individual drug. To this end our Committee has labored, and, thus far, with most flattering prospects of brilliant success. Papers, bearing upon these classes of subjects are in course of preparation by physicians selected from among those best qualified for the work, and others, equally distinguished in the various departments, have consented to take leading parts in the discussions of these papers.

In order to correct a misapprehension, it may be stated that the object of the Committee is to serve the Congress, not to control it. Undoubtedly the Congress will adopt and enforce rules of its own—those governing the reception and discussion of essays included. This Committee does not deem itself authorized to reject any paper that may be offered on any medical or surgical subject whatsoever. Its object is to *in*clude papers of a certain character, but not to *ex*clude anything. All essays, whether prepared at the instance of the Committee or as voluntary contributions, must be passed upon by the Congress or its delegated authority; but the Committee will probably recommend and urge that such of the essays as are more or less in harmony with the above-mentioned views shall take precedence of others, and it is quite likely that these will occupy nearly all the available time of the session.

Notice is hereby given that to insure the publication of the title of any paper in the "Annual Circular and Programme," said title must be in the hands of the undersigned on or before April 5th, and the paper itself should be sent as soon thereafter as practicable to the Chairman of the Committee, Dr. T. Y. Kinne, of Paterson, N. J., in order that provision may be made for its discussion.

Pemberton Dudley, M. D.,
*Sec. of the Com. and Genenal Secretary of the A. I. H.*
S. W. Cor. 15th and Master Sts.,
Philadelphia, Pa.

